# Catheter-related bloodstream infections with coagulase-negative staphylococci: are antibiotics necessary if the catheter is removed?

**DOI:** 10.1186/s13756-019-0474-x

**Published:** 2019-01-29

**Authors:** Ursula Patricia Hebeisen, Andrew Atkinson, Jonas Marschall, Niccolò Buetti

**Affiliations:** 1 Department of Infectious Diseases, University Hospital Bern, Freiburgstrasse, 3010 Bern, Switzerland; 2 Paediatric Pharmacology and Pharmacometrics, University of Basel Children’s Hospital, Basel, Switzerland

**Keywords:** Intravascular catheter, Central venous catheter, Coagulase-negative staphylococci, CRBSI

## Abstract

**Background:**

Catheter-related bloodstream infections (CRBSI) with coagulase-negative Staphylococci (CoNS) are a common source of hospital-acquired bloodstream infections. The main objective of this study was to elucidate the role of systemic antibiotic therapy in the setting of catheter removal in adult patients with CoNS-CRBSI.

**Methods:**

We conducted a retrospective cohort study on patients with CoNS-CRBSI diagnosed between 2008 and 2016 with follow-up for up to 12 months. The main inclusion criterion was a removed intravascular catheter with quantitative catheter tip culture growing CoNS and the same CoNS identified in the blood culture of a given patient. Outcomes were *non-resolved infection* (i.e. either presence of prolonged bacteremia or symptoms attributed to CoNS-CRBSI > 2 days after catheter removal), *recurrence*, *mortality* and *length of hospitalization* after catheter removal. We compared outcomes between a group with antibiotic treatment prescribed according to current IDSA guidelines (≥5 days, “treatment” group) and a “no-treatment” group.

**Results:**

Our study population comprised 184 CoNS-CRBSI episodes. Seventy-six percent received antibiotic treatment ≥5 days, while 17% did not receive therapy. *Non-resolved infections* were absent from the patients who did not receive antibiotics. Severe neutropenia, hematologic cancer and immunosuppression were significantly more frequent in the treatment group. The subgroup analysis with 32 matched pairs showed no significant difference in frequency of non-resolved infection (0% in the no-treatment vs 15.6% in the ≥5 days treatment group, *p* = 0.06). The remaining outcomes were similar in the two groups.

**Conclusions:**

Our findings indicate that withholding antimicrobial therapy in CoNS-CRBSI is neither associated with short-term complications nor with long-term recurrences.

**Electronic supplementary material:**

The online version of this article (10.1186/s13756-019-0474-x) contains supplementary material, which is available to authorized users.

## Background

Catheter-related bloodstream infections (CRBSI) are the leading subset of hospital-acquired bloodstream infections (BSI) [1]. In Europe, BSI can be found in 1–3.1 cases per 1000 patient-days, 60% of which are catheter-associated [1, 2]. Coagulase-negative staphylococci (CoNS) are the most frequent etiology of BSI, especially in the setting of catheter-related infections [2, 3]. CoNS-CRBSI and CoNS bacteremia increase the duration of hospitalization, intensive care unit (ICU) length of stay, morbidity and therapy-related costs [4–6]. However, CoNS are considered low-virulence microorganisms and data on outcomes in terms of mortality due to these infections have been conflicting [5, 7, 8].

Few studies have examined the role of antibiotic treatment in CoNS bacteremias. The heterogeneity of these studies precludes any conclusion regarding efficacy. A small study suggested that early antimicrobial treatment might reduce the clinical impact of CoNS-CRBSI [5], whereas other researchers concluded that administering adequate antibiotics was irrelevant for outcomes [9]. Another recent analysis postulated that adequate empirical therapy is not a requirement as long as the primary focus is removed [7]. Current guidelines question the need for treating these infections [10–12]. In particular, for uncomplicated CoNS-CRBSI, the Infectious Diseases Society of America (IDSA) guideline recommends antibiotic treatment for 5–7 days in the setting of catheter removal [10]. However, the same guideline highlights that some experts recommend no antibiotic therapy in patients without intravascular prosthetic material unless fever and/or bacteremia persist after catheter withdrawal, thus leaving the choice to treat or not to treat these infections open [10]. To our knowledge, there has never been a direct comparison of clinical outcomes in patients with CoNS-CRBSI treated with or without antibiotic agents. The main objective of this study was to elucidate the role of systemic antibiotic therapy in the setting of catheter removal in adult patients with CoNS-CRBSI.

## Methods

### Study design and population

We performed a retrospective cohort study on patients with CoNS-CRBSI admitted to Bern University Hospital, a 950-bed tertiary care hospital in Bern, Switzerland.

Patients with CoNS-CRBSI were identified by matching positive blood cultures and catheter tip data extracted from the clinical microbiology lab database at the University of Bern. The inclusion criteria for this study were the following: (1) age ≥ 18 years, (2) a removed short- or long-term intravascular catheter with a positive quantitative catheter tip culture, (3) the same CoNS (with an identical antibiogram) isolated from a blood culture drawn 7 days before to 2 days after catheter removal and (4) admission to the hospital between 1 January 2008 and 31 December 2016. Patients with *Staphylococcus lugdunensis* infection were excluded.

### Episode definition

In general, an *episode* of CoNS-CRBSI had to fulfill the IDSA criteria for definite CRBSI [10]: the same CoNS grows from at least 1 percutaneous blood culture and from the catheter tip, with a bacterial count of ≥10^3^ colony forming units (CFU) [13]. Moreover, patients having solely positive blood cultures drawn from the catheter hub were only included if (1) local symptoms were documented (e.g., redness or swelling) or (2) systemic symptoms (e.g., fever or chills) without another clinical focus were observed, thus qualifying at least for a complicated “exit-site infection” or “possible CRBSI” [10]. CRBSI episodes diagnosed by differential time-to-positivity were not considered for this analysis, as catheter removal was a selection criterion for our study. Moreover, additional culture tip reports of another catheter tip with CoNS in the same patient within 7 days after catheter removal were interpreted as *duplicate* and therefore excluded.

### Clinical, catheter and microbiological data

Data on included patients were obtained through review of their electronic medical records using a standardized data collection tool. For each episode demographic features, site of CRBSI onset, department at time of onset, underlying chronic diseases including the Charlson Comorbidity Index (CCI) [14] and immunosuppression (due to medication, chemotherapy or illness), and presence of orthopedic hardware or intravascular prosthetic material (other than intravascular catheters) were recorded. Clinical, catheter and microbiological data collected included: local exit-site infection signs, systemic symptoms (e.g., fever), severe neutropenia (leucocyte count < 0.5 G/L), type of catheter, catheter site and dwell-time, number of blood cultures drawn during an episode, number of positive blood cultures and resistance patterns of CoNS.

The antibiotic therapy was considered adequate if it included at least one antibiotic (e.g., vancomycin) to which the isolate was susceptible and the treatment was initiated no later than 48 h after catheter removal. In a subanalysis, we compared episodes treated according to current guidelines [3, 10–12, 15] versus episodes without antibiotic treatment.

### Follow-up and clinical outcomes

The patient charts during hospitalization and after catheter removal were carefully reviewed. All microbiological data of a patient with CoNS-CRBSI were reviewed for a 12-month follow-up period.

It is still debated if mortality represents a reasonable outcome measure for CoNS-CRBSI. In contrast to *S. aureus* and Gram-negative bacteremia [16], there is no standardized definition in the literature for treatment success of these infections. Therefore, we modified a previously used definition to determine the primary outcome [17]: A *non-resolved infection* was defined as prolonged bacteremia (persistence of positive blood culture with CoNS) or the presence of symptoms attributed to CoNS-CRBSI (e.g., septic thrombosis, abscess) starting more than 2 days after the catheter removal.

Secondary outcomes were *hospital mortality*, *ICU admission* during an episode, *length of hospital stay* after catheter removal, *mortality during follow-up* and *recurrence*. *Recurrence* was defined as the occurrence of the same CoNS (bacteremia or growth of CoNS in a sterile site), within 1 week to 12 months after the date of the initial bacteremia or until death (modified from Raad and colleagues [17]). We also collected adverse events attributed to the antibiotic therapy.

The study was approved by the regional ethics committee (project number 2017–01827).

### Statistical analysis

Group comparisons for continuous variables were carried out using the Wilcoxon rank test, whereas for categorial variables the χ2 or Fisher’s exact test were used.

As primary analysis model, we fitted logistic regression models with the dependent variable being the presence of non-resolved infection, and independent variables treatment group (antibiotic treatment ≥5 days vs no treatment), an indicator variable for age group, gender, CCI, catheter type, department, severe neutropenia during the episode, fever, and exit-site infection signs.

We performed a *post-hoc* subgroup analysis to attempt to adjust for inherent imbalance between the patient populations in the two groups. We used both inverse probability weighting (results not shown) and propensity score matching to create a subgroup with 32 matched pairs, 32 with treatment and 32 without treatment. To calculate the scores, we fitted a logistic regression model with treatment group as dependent variable, and an extended set of independent variables to enable us to use all available information for the matching process. We then used nearest neighbor matching to determine the closest pairs. We visually checked the appropriateness of the pairings by plotting the first two principal components from a principal components analysis using the same extended set of covariates (see Additional file 1: Figures S1-S2). Once this process was complete, we again compared the groups using both descriptive statistics and by fitting the primary analysis model to this subgroup. Throughout, *p*-values < 0.05 were considered statistically significant. All analyses were conducted using SPSS (Version 25) or R (Version 3.4.2).

## Results

**Fig. 1:**
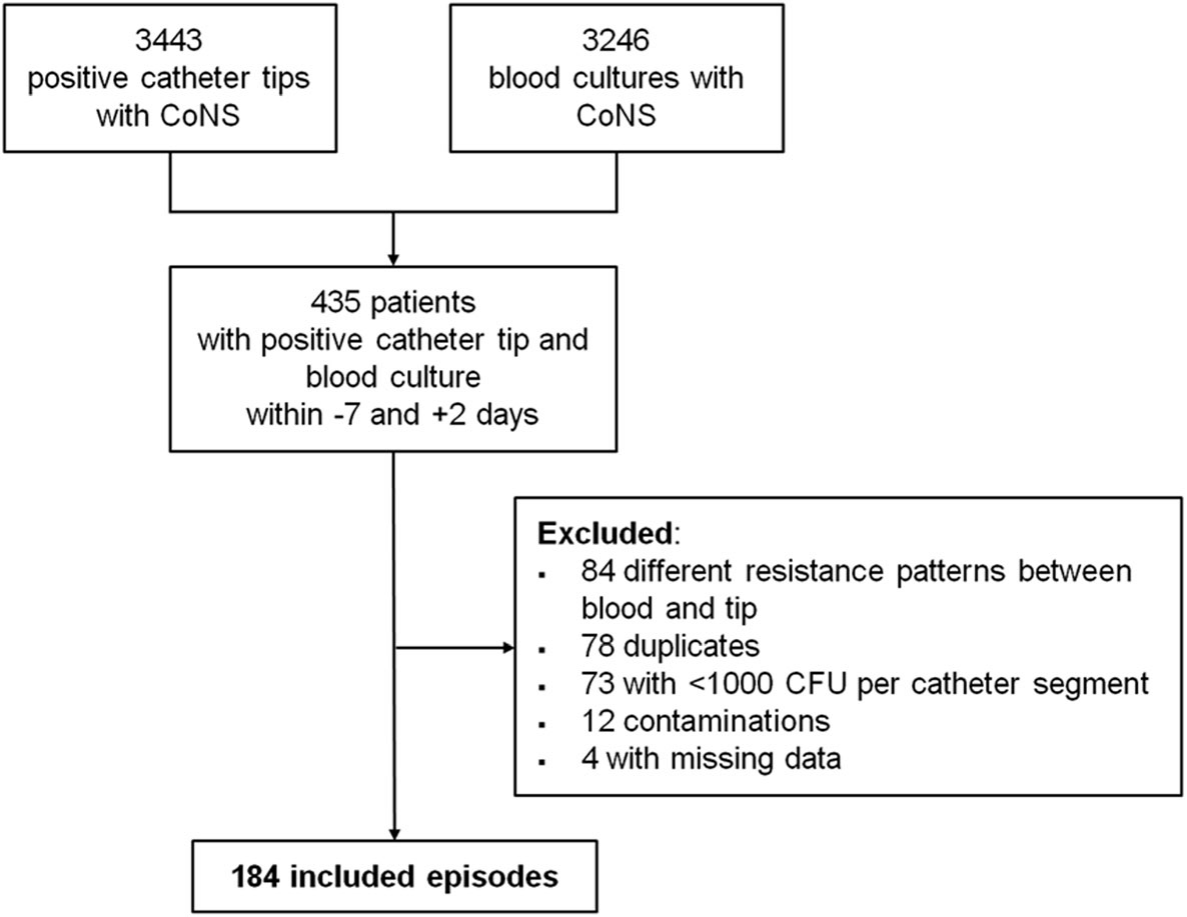
Data sources. CoNS, coagulase-negative Staphylococci; CFU, colony forming units

During the study period, a total of 3′443 positive catheter tips and 3′246 positive blood cultures with CoNS were identified (see Fig. 1). Of these, 435 patients presented a concurrent bacteremia with the same CoNS identified 7 days before to 48 h after catheter removal. Eighty-four CoNS episodes were excluded because they revealed different resistance patterns, 78 were duplicates and 73 had microbial counts per catheter segment that were considered too low. Our study population thus comprised 184 CoNS-CRBSI episodes.

### Patient characteristics and antibiotic therapy

The clinical characteristics of patients with CoNS-CRBSI (*n* = 184) are illustrated in Table 1. The median age was 61 years and patients were predominantly male (70%). Ninety-six percent (177) of CoNS-CRBSI were hospital-acquired; 41% (75) and 21% (38) were detected in the hemato-oncological and internal medicine departments, respectively. Comorbidities such as malignancy (109, 59%), immunosuppression (98, 53%) and diabetes mellitus (40, 22%) were frequently encountered. The mean Charlson Comorbidity Index was 4 (range 0–12). Forty-nine patients (27%) had orthopedic hardware or intravascular prosthetic material in place at the time of the index hospital admission. Of the 184 patients with CoNS-CRBSI, 71% (130) were diagnosed with fever, 35% (64) presented local signs and 42% (78) were severely neutropenic. Ninety-two percent of catheters (170) were short-term intravascular catheters, most of them CVCs (150, 82%).

**Table 1:** Patient characteristics: all, with adequate antibiotic treatment according to current guidelines (≥5 days), and without antibiotic treatment

Characteristic	Total* (*n* = 184)	Treatment ≥5 days (*n* = 140)	No treatment (*n* = 32)	*p* value
Age, years, median (IQR)	61 (51–67)	62.5 (51–67.5)	57.5 (52–63)	0.254
Sex, male	128 (70%)	96 (69%)	24 (75%)	0.616
BMI, kg/cm^2^, median (IQR)	26 (22–30)	26 (22–30)	25 (22–28.5)	0.535
Days in hospital, median (IQR)	29.5 (21–44)	31 (22–44)	28 (20–53.5)	0.783
Hospital-acquired CRBSI	177 (96%)	134 (96%)	32 (100%)	0.510
Department at time of diagnosis				
ICU	37 (20%)	24 (17%)	10 (31%)	0.118
Surgery	34 (19%)	26 (19%)	8 (25%)	0.563
Internal medicine	38 (21%)	22 (16%)	12 (38%)	0.011
Hemato-oncology	75 (41%)	68 (49%)	2 (6%)	< 0.001
Comorbidities				
Malignancy	109 (59%)	91 (65%)	11 (34%)	0.003
Hematologic cancer	82 (45%)	74 (53%)	2 (6%)	< 0.001
Solid cancer	27 (15%)	17 (12%)	9 (28%)	0.045
Immunosuppression	98 (53%)	85 (61%)	5 (16%)	< 0.001
Chronic pulmonary disease	22 (12%)	15 (11%)	4 (13%)	1.000
Congestive heart failure	14 (8%)	10 (7%)	4 (13%)	0.521
Renal failure	22 (12%)	11 (8%)	7 (22%)	0.044
Cerebrovascular disease	11 (6%)	10 (7%)	1 (3%)	0.662
Diabetes mellitus	40 (22%)	28 (20%)	10 (31%)	0.251
CCI, median (range)	4 (0–12)	4 (0–12)	4 (1–10)	0.609
Any surgical treatment	74 (40)	52 (37%)	18 (56%)	0.074
Orthopedic hardware or intravascular prosthetic material				
Any device	49 (27%)	40 (29%)	8 (25%)	0.851
Orthopedic hardware	25 (14%)	19 (14%)	5 (16%)	0.984
Intravascular prosthetic material	25 (14%)	21 (15%)	4 (13%)	0.933
Clinical findings				
Exit-site infection signs	64 (35%)	56 (40%)	3 (9%)	0.002
Fever (> 38.2 °C)	130 (71%)	107 (76%)	17 (53%)	0.015
Septic shock	4 (2%)	3 (2%)	0 (0%)	0.931
Severe neutropenia°	78 (42%)	71 (51%)	1 (3%)	< 0.001
Catheter characteristics				
Short-term catheter	170 (92%)	129 (92%)	30 (94%)	1.000
CVC	150 (82%)	116 (83%)	25 (78%)	0.709
Dwell time, median (IQR)	12 (9–17)	12 (9–17)	14 (9.5–20)	0.455
Long-term catheter	14 (8%)	11 (8%)	2 (6%)	1.000
Catheter site: jugular vein	138 (75%)	103 (74%)	26 (81%)	0.497
Microbiological data				
Positive blood cultures, median (IQR)	2 (1–3)	2 (2–3)	1 (1–2)	< 0.001
Total of blood cultures drawn, median (range)	4 (3–7)	5 (3–7)	2.5 (2–5)	0.001
≥ 10′000 CFU on catheter tipª	124 (67%)	93 (66%)	22 (69%)	0.965
CoNS resistant to oxacillin	143 (78%)	104 (74%)	30 (94%)	0.031
Antimicrobial therapy				
Vancomycin	135 (73%)	126 (90%)	0 (0%)	
Duration of adequate therapy, median (IQR)	10 (5–15)	13 (8–18)	0 (0)	

Abbreviations: *IQR* interquartile range, *BMI* body mass index, *ICU* intensive care unit, *CCI* Charlson comorbidity index, *CVC* central venous catheter, *CFU* colony forming units

All values expressed as no. (%), unless otherwise indicated.*including all patients with ≥1 day treatment or no treatment. **°** Severe neutropenia was defined as a leucocyte count < 0.5 G/L **ª** the remaining patients had a 1′000 CFU growth on their removed catheter tips

**Fig. 2:**
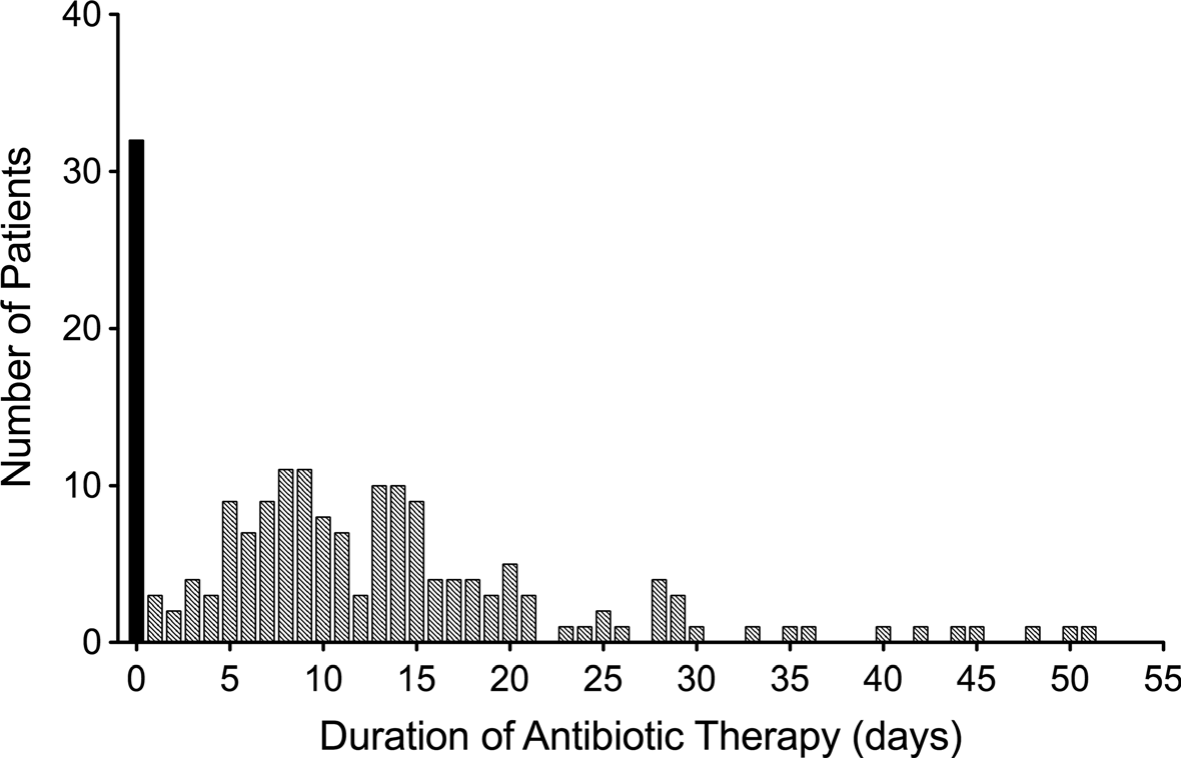
Distribution of the duration of antibiotic therapy (*n* = 184)

Figure 2 shows the distribution of treatment duration in our study group. Eighty-three percent of patients (152) received adequate antibiotic therapy (Additional file 1: Table S1) and 76% (140) were treated according to the current IDSA guidelines (i.e., at least 5–7 days). The median duration of treatment was 10 (IQR, 5–15) days. Vancomycin was the most frequently administered adequate antibiotic agent (135, 73%).

### Patients without antibiotic therapy and comparison with patients who received treatment ≥5 days

Two groups of patients were created, one that received adequate therapy for ≥5 days and the other without antibiotic therapy. Among patients without antibiotic treatment (32, 17%, “no treatment” group in Table 1), CRBSI were most frequently observed in internal medicine departments (12, 38%). The median Charlson Comorbidity Index was 4, 34% (11) of patients had a malignancy, and 25% (8) had orthopedic hardware or intravascular prosthetic material in situ on admission. Severe neutropenia was infrequently observed (1, 3%) in this group.

In general, the two groups presented similar baseline characteristics (Table 1). Similar percentages regarding the presence of prosthetic material and catheter characteristics were observed in both groups. However, CoNS-CRBSI in the “no treatment” group were less frequently observed in the hemato-oncologic department (6% vs 49% in the “treatment” group, *p* < 0.001), had less often hematologic cancer (6% vs 53%, *p* < 0.001), were less severely neutropenic (3% vs 51%, *p* < 0.001), and presented both fewer local (9% vs 40%, *p* = 0.002) and systemic signs (53% vs 76%, *p* = 0.015).

### Evaluation of outcomes

The mean follow-up time was 234 (SD ±155.1) days (Table 2). Of the 184 patients with CoNS-CRBSI, a non-resolved infection was observed in 30 patients (16%), with septic thrombosis being the most frequently observed complication (25, 14%). Recurrences were infrequently detected (8, 4%; all bacteremias) and no prosthetic material infections with CoNS were observed during the entire follow-up. Focusing on raw group proportion comparisons, none of the patients without antibiotic therapy experienced a non-resolved infection (0% infections in the no-treatment group vs 22% in those who received treatment according to guidelines, *p* = 0.01). The subgroup analysis utilized propensity scores and nearest neighbor matching to compare groups of *similar* patients with and without antibiotic treatment. The rates of outcomes approached each other after propensity score matched adjustment in terms of the non-resolved infection (0% vs 16%, *p* = 0.06; Table 3 and Additional file 1: Figures S1-S2). The majority of the 32 patients chosen for these analyses were non-neutropenic (31, 96.9%).

**Table 2:** Outcomes by duration of treatment (≥5 days vs. no treatment)

Characteristic	All patients^a^ (*n* = 184)	Treatment ≥5 days (*n* = 140)	No treatment (*n* = 32)	*p*-value
Primary outcome				
Non-resolved infection	30 (16%)	29 (21%)	0 (0%)	0.010
Septic thrombosis	25 (14%)	24 (17%)	0 (0%)	0.025
Prolonged bacteremia	4 (2%)	4 (3%)	0 (0%)	0.751
Abscess	2 (1%)	2 (1%)	0 (0%)	1.000
Secondary oucomes				
Recurrence (bacteremia)	8 (4%)	8 (6%)	0 (0%)	0.358
ICU admission	18 (10%)	15 (11%)	1 (3%)	0.111
Days to discharge, median (IQR)	13 (7–21)	15 (8–21)	11.5 (5–32)	0.253
Hospital mortality	22 (12%)	12 (9%)	6 (19%)	0.169
Mortality during follow-up	38 (21%)	26 (19%)	8 (25%)	0.563
Side effects	3 (2%)	3 (2%)		
Days of follow-up, mean (±SD)	234.2 (±155.1)	241.2 (±152.3)	232.4 (±161.2)	

Abbreviations: *ICU* intensive care unit, *IQR* interquartile range, *SD* standard deviation

All values expressed as no. (%), unless otherwise indicated. ^a^including all patients with ≥1 day treatment or no treatment

**Table 3:** Group comparisons following the nearest neighbor matching process

Characteristic	Treatment ≥5 days (*n* = 32)	No Treatment (*n* = 32)	*p*-value
Age mean (±SD)	58.0 (±15.7)	57.3 (±11.5)	0.892
Sex (mean)	26 (81%)	24 (75)	0.762
ICU	11 (34%)	10 (31%)	1.000
Internal medicine	11 (34%)	12 (38%)	1.000
Malignancy	13 (41%)	11 (34%)	0.796
Hematologic cancer	3 (9%)	2 (6%)	1.000
Immunosuppression	5 (16%)	5 (16%)	1.000
Renal failure	5 (16%)	7 (22%)	0.749
CCI, mean	4.3	4.1	0.109
Any surgical treatment	21 (66%)	18 (56%)	0.608
Orthopedic hardware or intravascular prosthetic material	10 (31%)	8 (25%)	0.781
Fever (> 38.2 °C)	19 (59%)	17 (53%)	0.801
Catheter type: short-term	29 (91%)	30 (94%)	1.000
Primary endpoint (Non-resolved Infection)	5 (16%)	0 (0%)	0.062

Abbreviations: *SD* standard deviation, *ICU* intensive care unit, *CCI* Charlson Comorbidity Index

All values expressed as no. (%), unless otherwise indicated

On the basis of univariate analyses, the secondary outcomes were similar in the two comparison groups (Table 2). In particular, no recurrence was observed in the “no treatment” group. Side effects due to antibiotic therapy were observed in only three of 32 patients (2%).

## Discussion

Our findings indicate that patients not receiving antibiotics for CoNS-CRBSI after catheter removal experience a similarly low rate of complications as patients receiving ≥5 days of antibiotic therapy. This conclusion may probably only be applied to non-neutropenic patients given our results, which are supported by a neighbor analysis and a principal components analysis for the primary outcome.

To date, there are no well-designed and adequately powered trials to compare the role of antibiotic therapy in CoNS-CRBSI following catheter removal. Accordingly, practice often relies on prescriber preference and individual experience. The current IDSA guideline on catheter-related infections suggests prescribing antibiotic therapy for at least 5 to 7 days after catheter removal [10]. Other guidelines or reviews support similar recommendations [3, 11, 12, 15]. Alternatively, patients with CoNS-CRBSI can be followed without antibiotic administration if the patients have neither orthopedic hardware nor intravascular prosthetic material in place. This strategy is based on expert opinion [10] and current recommendations (2009) are not based on relevant clinical evidence. Unfortunately, little progress has been made since that time to improve the evidence base regarding antibiotic administration for CoNS-CRBSI.

In a retrospective study, Raad et al. concluded that prolonged antibiotic treatment was not associated with better resolution of bacteremia or significantly lower rate of recurrence. However, in Raad’s study patients without antibiotics were not separately analyzed [17]. In an observational case-control study including patients with and without CoNS bacteremia, Molina et al. found an association between mortality and delay of appropriate antibiotic treatment. However, worse outcomes related to inappropriate empirical treatment were observed irrespective of whether patients had CoNS-BSI or not. A sub-analysis performed in only those patients with CoNS bacteremia showed that appropriate empirical treatment or delay of appropriate treatment did not affect outcomes [5]. Recently, Park et al. concluded that inappropriate empirical therapy does not lead to poor outcomes in CoNS-CRBSI bacteremia [7]. The retention of an eradicable focus such as intravascular catheter, however, might adversely affect outcomes in CoNS bacteremia. Moreover, that study population also included bacteremic patients without a CoNS-CRBSI thus limiting the generalizability of their results to intravascular catheter infections [7]. Of note, all these studies included patients with catheter retention and none compared patient characteristics between the different groups in the setting of catheter removal as we did here. Our study clearly supports the option of following patients with CoNS-CRBSI without administering antibiotic treatment by providing the first direct comparison with the standard approach, antibiotic treatment. Interestingly, the presence of foreign bodies (e.g., orthopedic hardware or intravascular prosthetic material) did not influence the rate of late recurrences. To our knowledge, only one study has ever assessed the occurrence of recurrent CoNS-CRBSI [17], revealing that patients with catheter retention were more likely to suffer from infection recurrence. However, 1) only bacteremias were reviewed without considering other metastatic foci, and 2) the role of antibiotics was not assessed in a sub-analysis. Reducing antibiotic use, in particular vancomycin, may be effective in decreasing both several adverse effects in the short term [18, 19] and antibiotic resistance in the long term [20, 21].

Our study has several limitations. This was a small, retrospective, single-center study and the results may not be applicable to patients with severe neutropenia. However, the population included in the nearest neighbor analysis also included very ill patients and therefore our conclusions may be applied to a variety of patient settings. Moreover, patients receiving ≥5 days of antibiotic therapy showed higher risk of complications. Following matching, this difference was reduced, without statistical significance at the 5% level. The remaining difference might be explained by the presence of unmeasured confounders. Furthermore, patients without significant growth of CoNS on the catheter tip as well as CoNS-CRBSI diagnosed by differential time-to-positivity (without tip culture), were not included, which may have led to an underestimation of the total burden of CoNS-CRBSI. On one hand, no molecular typing was performed for recurrences, which can lead to overestimation of this specific outcome; on the other hand, patients were not on defined clinical and follow-up protocols, which may have led to an increased number of lost-to-follow-up patients.

## Conclusions

These limitations notwithstanding, our study clearly suggests that managing CoNS-CRBSI in non-neutropenic patients without clinical evidence of local symptoms solely with catheter removal appears to be an option with neither short-term complications nor long-term recurrences, even if orthopedic hardware or intravascular prosthetic material remain in place. To verify our findings and ensure patient safety, further investigations in prospective randomized trials are needed.

## Additional file

Additional file 1: **Figure S1** Distribution of propensity scores post matching process. **Figure S2.** Plot of the first two principal components with the full data set to determine the appropriateness of the matches from the nearest neighbor process. **Table S1.** Characteristics and outcome of patients with antibiotic treatment (≥1 day) vs. patients without antibiotic treatment. (PDF 332 kb)

## Abbreviations

CCI: Charlson Comorbidity Index
CoNS: Coagulase-negative staphylococci
CRBSI: Catheter-related bloodstream infections
ICU: Intensive Care Unit
IDSA: Infectious Diseases Society of America
