# Supplementary material for: Catheter-related bloodstream infections with coagulase-negative staphylococci: are antibiotics necessary if the catheter is removed?
Source: Antimicrob Resist Infect Control. 2019 Jan 29;8:21. doi: 10.1186/s13756-019-0474-x (PMC6352346; doi:10.1186/s13756-019-0474-x)
Supplement: Supplementary file 1 — Figure S1 Distribution of propensity scores post matching process. Figure S2. Plot of the first two principal components with the full data set to determine the appropriateness of the matches from the nearest neighbor process. Table S1. Characteristics and outcome of patients with antibiotic treatment (≥1 day) vs. patients without antibiotic treatment. (PDF 332 kb) [file 13756_2019_474_MOESM1_ESM.pdf]

Supplementary appendix

Figure S1: Distribution of propensity scores post matching process;

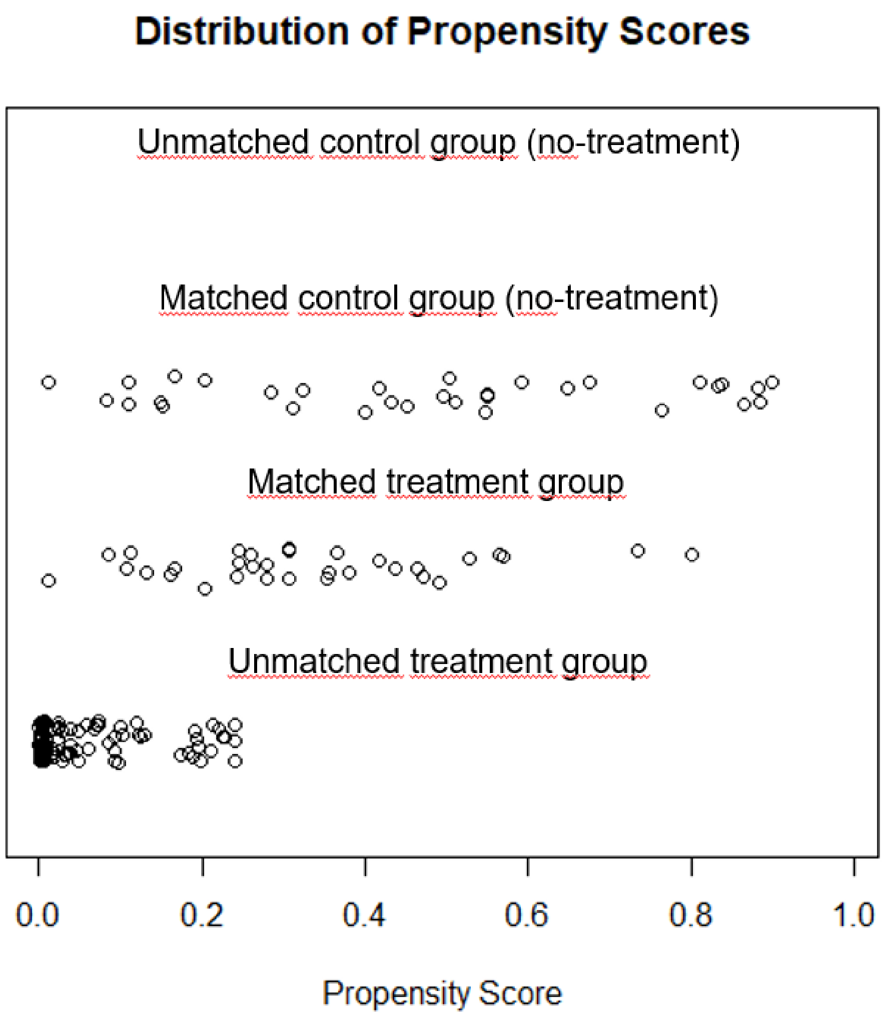

Note: In Figure S1 the calculated propensity score for the matched patients is plotted. It illustrates that the matching process found similar patients in both groups based on the scores.

Figure S2: Plot of the first two principal components with the full data set to determine the appropriateness of the matches from the nearest neighbor process.

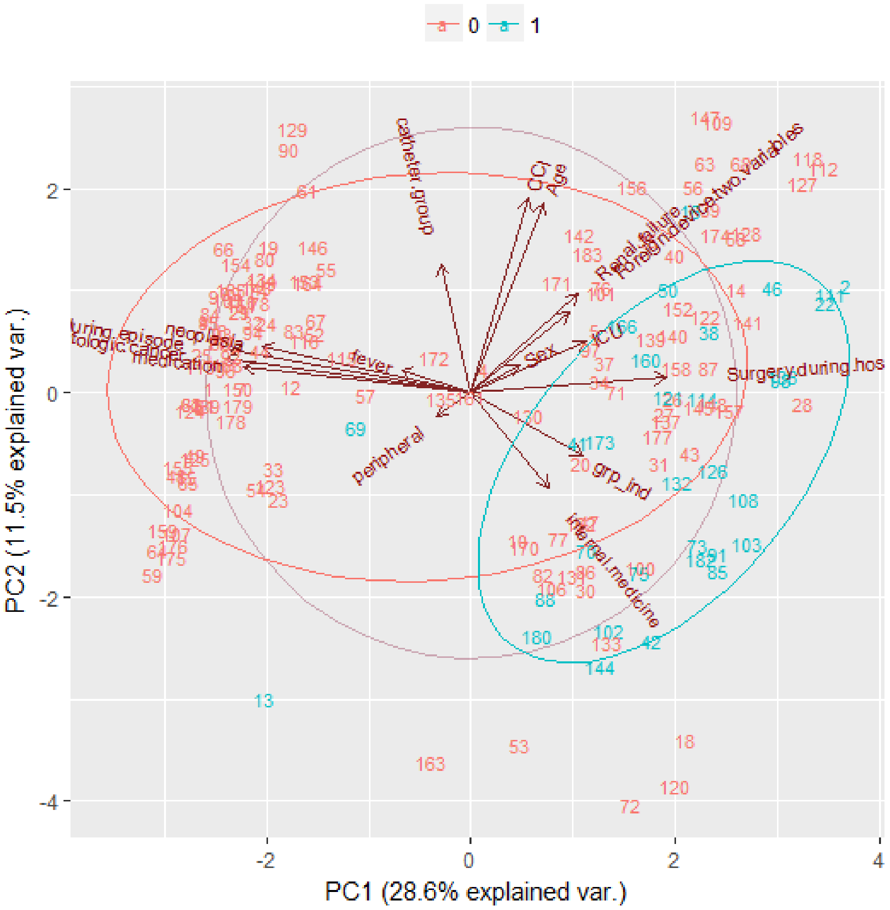

Note: Following the matching process we wanted a visual check to make sure the process did indeed find comparable pairs. We performed a principle components analysis using the same set of variables as for the primary analysis. Figure S2 plots the first two components (PC1 on the horizontal axis, PC2 on the vertical axis) which account for approximately 40% of the total variability. Each patient is annotated with the patient number. Those on and off. Treatment are colored pink (on = 0) and blue (off = 1), with the ellipses indicating possible groupings as suggested by the principle components package in R. Here the principle components analysis summarizes all the variables into two dimensions, and hence the arrows show the increasing magnitude for each variable. For example, in bottom right there is the “internal medicine” arrow, indicating that those patients in the bottom right corner are more likely to be from the internal medicine department. If we consider the blue ellipse, which corresponds to most of the 32 “off treatment” patients, we note that in most cases there are corresponding likely pairs from the “on treatment” group in pink. We cross-checked these with the matching pairs suggested by the propensity score method, and these were in most cases the proposed matches. We note that there are some clusters of “off treatment patients” without a close “on treatment” match in the figure (e.g. bottom right, patients 108, 103, 73, 91, 18, 85). These correspond to the patients not matched on the far right of Figure S1.”

Table A: Characteristics and outcome of patients with antibiotic treatment (≥1 day) *vs*. patients without antibiotic treatment

Characteristic	Treatment (n=152)	No treatment (n=32)	p-value
**Age, years, mean (±SD)**	58.2 (±14.1)	57.5 (±11.5)	0.243
**Sex, male**	104 (68%)	24 (75%)	0.531
**BMI, kg/cm^2, median (range)**	26 (14-63)	25 (17-39)	0.457
**Days in hospital, mean (±SD)**	35.9 (±22.0)	44.3 (±46.5)	0.980
**CoNS-CRBSI hospital acquired**	145 (95%)	32 (100%)	0.607
**Department at time of diagnosis**			
ICU	27 (18%)	10 (31%)	0.093
Surgery	26 (17%)	8 (25%)	0.319
Internal medicine	26 (17%)	12 (38%)	0.015
Hemato-oncology	73 (48%)	2 (6%)	<0.001
**Comorbidities**			
Malignancy	98 (65%)	11 (34%)	0.002
Hematologic cancer	80 (53%)	2 (6%)	<0.001
Solid cancer	18 (12%)	9 (28%)	0.027
Immunodeficiency	93 861%)	5 (16%)	<0.001
Chronic pulmonary disease	18 (12%)	4 (13%)	1.000
Congestive heart failure	10 (7%)	4 (13%)	0.271
Renal failure	15 (10%)	7 (22%)	0.072
Cerebrovascular disease	10 (7%)	1 (3%)	0.692
Diabetes mellitus	30 (20%)	10 (31%)	0.162
CCI, median (range)	4 (0-12)	4 (1-10)	0.609
Any surgical treatment	56 (37%)	18 (56%)	0.049
**Orthopedic hardware or intravascular prosthetic material**			
Any device	41 (27%)	8 (25%)	1.000
Orthopedic hardware	20 (13%)	5 (16%)	0.777
Intravascular prosthetic material	21 (14%)	4 (13%)	1.000
**Clinical findings**			
Redness	61 (40%)	3 (9%)	<0.001
Fever (>38.2°C)	113 (74%)	17 (53%)	0.031
Septic shock	4 (3%)	0 (0%)	1.000
Neutropenia	77 (51%)	1 (3%)	<0.001
**Catheter characteristics**			
Short-term catheter	140 (92%)	30 (94%)	1.000
CVC	125 (82%)	25 (78%)	0.618
Long-term catheter	12 (8%)	2 (6%)	1.000
Catheter site: jugular vein	112 (74%)	26 (81%)	0.501
**Microbiology data**			
Positive blood cultures, median (range)	2 (1-6)	1 (1-7)	<0.001
Total of blood cultures drawn, median (range)	5 (1-15)	2.5 (1-13)	0.002
>1000 CFUs on catheter tip	102 (67%)	22 (69%)	1.000
CoNS resistant to oxacillin	113 (74%)	30 (94%)	0.018
**Antimicrobial therapy**			
Vancomycin	135 (89%)	0 (0%)	
Duration of adequate therapy, mean (±SD)	14.2 (±10.1)	0.0 (±0)	

Table A: continued

**Outcomes**			
**Non-resolved infection**	30 (20%)	0 (0%)	0.003
Septic thrombosis	25 (16%)	0 (0%)	0.009
Prolonged bacteremia	4 (3%)	0 (0%)	1.000
Abscess	2 (1%)	0 (0%)	1.000
**Recurrence (bacteremia)**	8 (5%)	0 (0%)	0.354
**ICU admission**	17 (11%)	1 (3%)	0.312
**Side effects (antibiotics)**	3 (2%)	0 (0%)	1.000
**Days to discharge, mean (±SD)**	17.6 (±15.8)	19.2 (±20.7)	0.444
**Death in hospital**	16 (11%)	6 (19%)	0.228
**Death during follow-up**	30 (20%)	8 (25%)	0.481
**Days of follow-up, mean (±SD)**	234.6 (±154.3)	232.4 (±161.2)	0.955

All values expressed as no. (%), unless otherwise indicated.
Abbreviations: SD, standard deviation; BMI, body mass index; CoNS-CRBSI, coagulase-negative Staphylococci catheter-related bloodstream infection; ICU, intensive care unit; CCI, Charlson comorbidity index; CVC, central venous catheter; CFU, colony forming units
